# Path following control of planar snake robots using virtual holonomic constraints: theory and experiments

**DOI:** 10.1186/s40638-014-0003-6

**Published:** 2014-08-28

**Authors:** Ehsan Rezapour, Kristin Y Pettersen, Pål Liljebäck, Jan T Gravdahl, Eleni Kelasidi

**Affiliations:** Department of Engineering Cybernetics, Norwegian University of Science and Technology (NTNU), Trondheim, 7491 Norway; Department of Applied Cybernetics, SINTEF ICT, Trondheim, 7465 Norway

**Keywords:** Path following, Snake robots, Biologically inspired robots, Virtual holonomic constraints

## Abstract

This paper considers path following control of planar snake robots using virtual holonomic constraints. In order to present a model-based path following control design for the snake robot, we first derive the Euler-Lagrange equations of motion of the system. Subsequently, we define geometric relations among the generalized coordinates of the system, using the method of virtual holonomic constraints. These appropriately defined constraints shape the geometry of a constraint manifold for the system, which is a submanifold of the configuration space of the robot. Furthermore, we show that the constraint manifold can be made invariant by a suitable choice of feedback. In particular, we analytically design a smooth feedback control law to exponentially stabilize the constraint manifold. We show that enforcing the appropriately defined virtual holonomic constraints for the configuration variables implies that the robot converges to and follows a desired geometric path. Numerical simulations and experimental results are presented to validate the theoretical approach.

## Introduction

Although wheels and legs are extensively used in mobile robots and are known as the conventional locomotion tools, we sometimes need more adaptable and flexible locomotion systems in order to carry out tasks in complex, narrow, and unstructured environments. In such situations, snake robots, which have significant adaptability and structural flexibility properties, are a potentially useful alternative to conventional types of mobile robots. Snake robots are relevant for applications where restriction of human involvement is important due to safety (e.g. in firefighting operations [1]) and in applications where human presence is impossible (e.g. in narrow pipe inspection tasks [2,3]).

This paper considers path following control of snake robots. In the path following problem, the goal is to ensure that the error between the system output and a desired geometric path is asymptotically less than any pre-specified constant, while guaranteeing a forward motion along the path and boundedness of the states of the controlled system [4]. This control problem is particularly challenging for snake robots. This is due to the fact that such mechanisms are generally hyper-redundant, i.e. they have a large degree of kinematic redundancy, and this gives rise to a complicated dynamical behaviour for the system. Moreover, snake robots are underactuated, i.e. they have fewer independent control inputs than degrees of freedom (DOF), and this complicates the control design for these robots.

## Background and literature review

In general, snake robots can be categorized into two classes: snake robots which are subject to nonholonomic velocity constraints and snake robots without nonholonomic velocity constraints. Path following control of both classes of snake robots has been considered in several previous works. The majority of these works consider snake robots with nonholonomic velocity constraints, which is inspired by the world’s first snake robot developed in 1972 [5]. Nonholonomic constraints are in the form of sideslip constraints on the links of the robot, i.e. where each link is constrained from moving sideways. These constraints allow the control input to be specified directly in terms of the desired propulsion of the snake robot, which is employed in [6-8] for computed torque control of the position and heading of snake robots with nonholonomic velocity constraints. In [9], position and path following controllers are proposed for the case where some, but not all, of the snake robot links are subject to sideslip constraints. These constrained links can be lifted from the ground, which give the system more DOF that can be utilized to follow a trajectory while simultaneously maintaining a high manipulability. Similar approaches are considered in [10], where strategies for sinus lifting during the lateral undulatory motion are proposed. In [11], a path following controller for a snake robot with nonholonomic velocity constraints is proposed, and Lyapunov analysis is employed in order to analyse the controller.

Path following control of snake robots without nonholonomic velocity constraints is only considered in a few previous works. In [12], path following control of swimming snake robots is achieved by moving the joints according to a predetermined gait pattern while introducing an angular offset in each joint to steer the robot to some desired path. Methods based on numerical optimal control are considered in [13] for determining optimal gaits during positional control of snake robots. In [14], a control strategy is proposed for sinus lifting during lateral undulation by solving a quadratic optimization problem. In [15], numerical simulations are used to study the properties of lateral undulation that are related to the optimality of motion of the snake robot. In [16,17], cascaded systems theory is employed to achieve path following control of a snake robot described by a simplified model. In this simplified model of the snake robot, the motion of the links is approximated as translational motion instead of rotational motion, which is valid for small joint angles. In [18], a dynamic feedback control law is proposed which controls the body shape and orientation of the snake robot. Controllability analysis of planar snake locomotion is presented in [19], and a controller for straight line path following control of snake robots is proposed and a Poincare map is investigated to prove that the resulting state variables of the snake robot, except for the position in the forward direction, trace out an exponentially stable periodic orbit.

## Research design and methodology

The contribution of this paper is a solution to the path following control problem for a snake robot without nonholonomic velocity constraints, by using virtual holonomic constraints, which is a particularly useful concept for control of oscillations (see, e.g. [20-24]). Using this approach, we constrain the state evolution of the system to an appropriately defined submanifold of the configuration space, which is called the constraint manifold. This manifold is defined based on the specified geometric relations among the generalized coordinates of the system which are called virtual holonomic constraints. The proposed feedback control law is designed to exponentially stabilize the constraint manifold, i.e. to enforce the virtual holonomic constraints, which allows the convergence of the snake robot to the desired path.

To our best knowledge, the application of model-based motion control approaches which rely on formal stability proofs for snake robots is very restricted in the previous literature. In particular, the only previous works which formally prove the stability of a path following controller for a snake robot without nonholonomic velocity constraints are presented in [17,18]. In [17], cascaded systems theory is used to stabilize a desired straight path for the position of the center of mass (CM) of a snake robot which is described based on simplified kinematic and dynamic models. In contrast with [17], in this paper, we consider a more complex and accurate model of the snake robot, where the motion of the links is not modelled based on the simplifying assumptions of [17]. Preliminary results of this paper are presented in [18], where the method of virtual holonomic constraints is used for path following control of a planar snake robot. In this paper, these results are extended with a new simulation study of a 11-link snake robot. Furthermore, we extend the results presented in [18] by an experimental investigation of the performance of the control approach. A back-to-back comparison between simulations and experimental results is given, in order to contribute to bridge the gap between advanced control theory and practice.

The paper is organized as follows. First, we derive the Euler-Lagrange equations of motion of the robot. Subsequently, we state the control design objectives. Afterwards, we use the virtual holonomic constraints approach for path following control design for the snake robot. Finally, we present the results of numerical simulations and real-time experiments which illustrate the performance of the theoretical control design.

## Methods

In this section, we derive the kinematic model along with the dynamic equations of motion of the snake robot in a Lagrangian framework. Moreover, we use partial feedback linearization to write the model in a simpler form for model-based control design.

**Figure 1:**
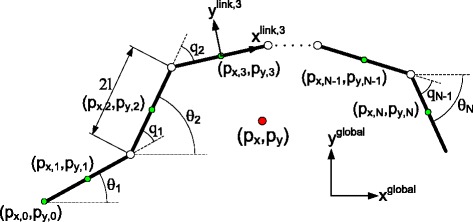
**An illustration of the** ***N*** **-link snake robot.** Kinematic parameters of the snake robot.

In order to perform control design, we need to write the governing equations of the system in an implementable way. This is often done by choosing a local coordinate chart and writing the system equations with respect to (w.r.t.) these coordinates. According to the illustration of the snake robot in Figure 1, we choose the vector of the generalized coordinates of the *N*-link snake robot as $x=\left [q_{1},q_{2},\ldots,q_{N-1},\theta _{N},p_{x},p_{y}\right ]^{T}\in \mathbb {R}^{N+2}$, where *q*_*i*_ with *i*∈{1,…,*N*−1} denotes the *i*th joint angle, *θ*_*N*_ denotes the head angle, and the pair (*p*_*x*_,*p*_*y*_) describes the position of the CM of the robot w.r.t. the global *x*−*y* axes. Since the robot is not subject to nonholonomic velocity constraints, the vector of the generalized velocities is defined as $\dot {x}=\left [\dot {q}_{1},\dot {q}_{2},\ldots,\dot {q}_{N-1},\dot {\theta }_{N},\dot {p}_{x},\dot {p}_{y}\right ]^{T} \in \mathbb {R}^{N+2}$. Using these coordinates, it is possible to specify the kinematic map of the robot. In this paper, we denote the first *N* elements of the vector *x*, i.e. (*q*_1_,…,*q*_*N*−1_,*θ*_*N*_), as the angular coordinates, and the corresponding dynamics as the angular dynamics of the system.

### The geometry of the problem

The (*N*+2)-dimensional configuration space of the snake robot is denoted as $\mathcal {Q}=\mathcal {S} \times \mathcal {G}$, which is composed of the shape space  and a Lie group  which is freely and properly acting on the configuration space. In particular, the shape variables, i.e. *q*_*a*_=(*q*_1_,…,*q*_*N*−1_), which define the internal configuration of the robot and which we have direct control on, take values in . Moreover, the position variables, i.e. *q*_*u*_=(*θ*_*N*_,*p*_*x*_,*p*_*y*_), which are passive DOF of the system, lie in . The velocity space of the robot is defined as the differentiable (2*N*+4)-dimensional tangent bundle of  as $T\mathcal {Q}=\mathbb {T}^{N} \times \mathbb {R}^{N+4}$, where $\mathbb {T}^{N}$ denotes the *N*-torus in which the angular coordinates live. The free Lagrangian function of the robot $\mathcal {L}:T\mathcal {Q} \rightarrow \mathbb {R}$ is invariant under the given action of  on . The coupling between the shape and the position variables causes the net displacement of the position variables, according to the cyclic motion of the shape variables, i.e. the *gait pattern*. Note that for simplicity of presentation, in this paper, we consider local representation of $T\mathcal {Q}$ embedded in an (2*N*+4)-dimensional open subset of the Euclidean space $\mathbb {R}^{2N+4}$.

### The forward kinematic map of the snake robot

Based on the kinematic parameters of the snake robot given in Figure 1, it is possible to write the coordinate representation of the forward kinematic map. The map between the absolute link angles *θ*_*i*_ and the relative joint angles *q*_*i*_ is given by

(1) $$ \theta_{i} = \sum\limits_{n=i}^{N-1}q_{n}+\theta_{N}  $$

The position of the CM of the *i*th link w.r.t. the global *x*-*y* axes can be, respectively, given as

(2) $$\begin{array}{@{}rcl@{}} p_{x,i}&=&p_{x,0}+2l\sum\limits_{j=1}^{i-1}\cos\theta_{j}+l\cos\theta_{i} \end{array} $$

(3) $$\begin{array}{@{}rcl@{}} p_{y,i}&=&p_{y,0}+2l\sum\limits_{j=1}^{i-1}\sin\theta_{j}+l\sin\theta_{i} \end{array} $$

where 2*l* denotes the length of each link, and (*p*_*x*,0_,*p*_*y*,0_) denotes the tail position (*cf.* Figure 1). The linear velocities of the CM of the *i*th link w.r.t. the global *x*-*y* axes can be found by taking the time derivative of (2)-(3) which gives

(4) $$\begin{array}{@{}rcl@{}} \dot{p}_{x,i}&=&\dot{p}_{x,0}-2l\sum\limits_{j=1}^{i-1}\sin\theta_{j} \dot{\theta}_{j}-l\sin \theta_{i}\dot{\theta}_{i} \end{array} $$

(5) $$\begin{array}{@{}rcl@{}} \dot{p}_{y,i}&=&\dot{p}_{y,0}+2l\sum\limits_{j=1}^{i-1}\cos\theta_{j} \dot{\theta}_{j}+l\cos \theta_{i}\dot{\theta}_{i} \end{array} $$

Since all the links have equal length and mass, the position of the CM for the whole structure of the robot is defined as

(6) $$\begin{array}{@{}rcl@{}} \left(p_{x},p_{y}\right) = \left(\frac{1}{N} \sum\limits_{i=1}^{N} p_{x,i},\frac{1}{N} \sum\limits_{i=1}^{N} p_{y,i}\right) \end{array} $$

To facilitate path following control of the CM of the snake robot, we replace the tail position (*p*_*x*,0_,*p*_*y*,0_) in (2)-(3) with the position of the CM of the robot (*p*_*x*_,*p*_*y*_) using the following change of coordinates:

(7) $$ p_{x,0}=p_{x}-\frac{1}{N}\sum\limits_{i=1}^{N}\left(2l\sum\limits_{j=1}^{i-1}\cos\theta_{j}+l\cos\theta_{i}\right)  $$

(8) $$ p_{y,0}=p_{y}-\frac{1}{N}\sum\limits_{i=1}^{N}\left(2l\sum\limits_{j=1}^{i-1}\sin\theta_{j}+l\sin\theta_{i}\right)  $$

Substituting (7)-(8) along with their time derivatives into (2)-(5) completes the derivation of the forward kinematic map of the snake robot w.r.t. the desired specified coordinate chart $(x,\dot {x})$.

### Equations of motion of the snake robot

The majority of the previous literature on snake robots and similar mobile multi-body robotic structures, such as eel-like robots, have derived the equations of motion of these robots with a Newton-Euler formulation, i.e. where the equations describing the linear and angular motion of individual links are written separately (see, e.g. [15,16]). This is due to the fact that it is usually not straightforward to integrate the anisotropic external dissipative forces, i.e. ground friction forces, acting on these complex robotic structures into their Euler-Lagrange equations of motion. However, ground friction forces have been proved to play a fundamental role in snake robot locomotion (see, e.g. [16]). In this paper, we derive the equations of motion of the snake robot in a Lagrangian framework, i.e. treating the robot as a whole and performing the analysis using a Lagrangian function, which is simple to follow and better suited for studying advanced mechanical phenomena such as elastic link deformations [25], which might be insightful for future research challenges on snake robots. Moreover, we integrate the anisotropic friction forces into these equations using the Jacobian matrices of the links, which gives a straightforward mapping of these forces for the equations of motion.

Snake robots are a class of *simple* mechanical systems, where the Lagrangian $\mathcal {L}\left (q_{a},\dot {x}\right)$ is defined as the difference between the kinetic energy $\mathcal {K}(q_{a},\dot x)$ and potential energy $\mathcal {P}(x)$ of the system [26]. Since the planar snake robot is not subject to any potential field, i.e. $-\nabla \mathcal {P}(x)=0$, we may write the Lagrangian equal to the kinetic energy, which is the sum of the translational and the rotational kinetic energy of the robot:

(9) $$  \mathcal{L}\left(q_{a},\dot{x}\right)=\mathcal{K}\left(q_{a},\dot{x}\right)=\frac{1}{2}m \sum\limits_{i=1}^{N}\left(\dot{p}_{x,i}^{2}+\dot{p}_{y,i}^{2}\right)+\frac{1}{2}J \sum\limits_{i=1}^{N}\dot{\theta}_{i}^{2}  $$

where *m* and *J* denote the uniformly distributed mass and moment of inertia of the links, respectively. Using the Lagrangian function (9), we write the Euler-Lagrange equations of motion of the controlled system as

(10) $$ \frac{d}{dt}\left[\frac{\partial\mathcal{L}\left(q_{a},\dot{x}\right)}{\partial\dot{x}_{i}}\right]- \frac{\partial\mathcal{L}\left(q_{a},\dot{x}\right)}{\partial{x}_{i}}=\left(B(x)\tau-\tau_{f}\right)_{i}  $$

where *i*∈{1,…,*N*+2}, $B(x)=\left [e_{j}\right ]\in \mathbb {R}^{(N+2)\times (N-1)}$ is the full column rank actuator configuration matrix, where *e*_*j*_ denotes the *j*th standard basis vector in $\mathbb {R}^{N+2}$. Moreover, $B(x)\tau \in \mathbb {R}^{N+2}$ with $\tau =\left [\tau _{1},\ldots, \tau _{N-1}\right ]^{T}\in \mathbb {R}^{N-1}$ stands for the generalized forces resulting from the control inputs. Furthermore, $\tau _{f}=\left [{\tau _{f}^{1}},\ldots,\tau _{f}^{N+2}\right ]^{T}\in \mathbb {R}^{N+2}$ denotes viscous and Coulomb friction forces acting on (*N*+2) DOF of the system. The controlled Euler-Lagrange equations (10) can also be written in the form of a second-order differential equation as

(11) $$ M\left(q_{a}\right)\ddot{x}+C\left(x,\dot{x}\right)\dot{x}=B(x)\tau-\tau_{f}  $$

where $M(q_{a})\in \mathbb {R}^{(N+2) \times (N+2)}$ is the positive definite symmetric inertia matrix, $C(x,\dot {x})\dot {x}\in \mathbb {R}^{N+2}$ denotes the generalized Coriolis and centripetal forces, and the right-hand side terms denote the external forces acting on the system. The fact that the inertia matrix is only a function of the directly actuated shape variables *q*_*a*_ is a direct consequence of the invariance of the Lagrangian function (9) w.r.t. the position variables. Moreover, since rank[*B*(*x*)]<dim(*x*), the system is underactuated. This underactuation represents the lack of direct control on the head angle and the position of the CM of the robot.

The dynamic model (11) perfectly agrees with the equations of motion which are derived based on the Newton-Euler formulation in previous works (see, e.g. [16]). In order to validate the model, in the last section of this paper, we present simulation results which are obtained using the dynamic model (11) together with experimental results for the locomotion of the robot which are obtained using a physical snake robot. The agreement between simulations and experiments shows that the dynamic model (11) accurately represents the motion of the robot.

### The ground friction model

In this subsection, both viscous and Coulomb friction models are used for capturing the essential properties of the anisotropic ground friction forces. For modelling the friction, we first define the rotation matrix for mapping from the global frame to the local frame of link *i* (*cf.* Figure 1) as

(12) $$\begin{array}{@{}rcl@{}} R_{i}= \left[ \begin{array}{cc} \cos\theta_{i} & -\sin\theta_{i} \\ \sin\theta_{i} & \cos\theta_{i} \\ \end{array} \right] \end{array} $$

Using (4)-(5) and (12), the velocities of the links in the local link frames can be written in terms of the velocities of the links in the global frame as

(13) $$\begin{array}{@{}rcl@{}} v^{\text{link},i}= \left[ \begin{array}{cc} v_{t}^{\text{link}, i} & v_{n}^{\text{link},i} \end{array} \right]^{T} ={R_{i}^{T}} \left[ \begin{array}{cc} \dot{p}_{x,i} & \dot{p}_{y,i} \end{array} \right]^{T} \end{array} $$

where $v_{t}^{\text {link},i}$ and $v_{n}^{\text {link},i}$ denote the linear velocity of the CM of the *i*th link in the tangential (along link *x*-axis) and normal (along link *y*-axis) direction of the link, respectively. The total friction force acting on link *i* is defined as the sum of the viscous and Coulomb friction forces, which are denoted by $f_{v_{i}}$ and $f_{c_{i}}$, respectively, as

(14) $$ f^{\text{link},i}= f_{c_{i}}+f_{v_{i}}  $$

Assuming equal friction coefficients for all the links, we write the model of the friction for each individual link *i* as

(15) $$ f_{c_{i}} = mg \left[\mu_{t}\text{sgn}{\left(v_{t}^{\text{link},i}\right)} \ \mu_{n}\text{sgn}{\left(v_{n}^{\text{link},i}\right)}\right]^{T}\!\! \in \mathbb{R}^{2}  $$

(16) $$ f_{v_{i}} = \left[c_{t} v_{t}^{\text{link},i} c_{n} v_{n}^{\text{link},i}\right]^{T} \in \mathbb{R}^{2}  $$

where *i*∈{1,…,*N*}, *m* denotes the mass of a link, *g* denotes the acceleration due to gravity, and *μ*_*t*_ and *μ*_*n*_ denote Coulomb friction coefficients in the tangential and normal direction of the link, respectively. Furthermore, *c*_*t*_ and *c*_*n*_ denote viscous friction coefficients in the tangential and normal direction of the link, respectively. Thus, we map the friction force acting on the *i*th link to the global *x*-*y* frame as

(17) $$ f_{\text{global}}^{\text{link},i}=R_{i} f^{\text{link},i}  $$

Finally, we can write *τ*_*f*_ in (11) as

(18) $$ \tau_{f}=\sum\limits_{i=1}^{N}\mathcal{J}_{i}^{T}(x)\, f_{\text{global}}^{\text{link}, i}  $$

where

(19) $$ \mathcal{J}_{i}^{T} (x)=\left[\!\frac{\partial\dot{p}_{x,i}}{\partial \dot{x}_{j}}, \frac{\partial\dot{p}_{y,i}}{\partial \dot{x}_{j}}\right]\in \mathbb{R}^{(N+2)\times 2},\qquad j\in \{1,\ldots,N+2\}  $$

denotes the transpose of the Jacobian matrix of the CM of the *i*th link.

#### **Remark1**.

As argued in [16], the motion of a snake robot with anisotropic viscous ground friction is qualitatively (but not quantitatively) similar as with anisotropic Coulomb friction. However, a viscous friction model is less complex w.r.t. control design and analysis. Accordingly, we employ a viscous friction model for the control design in this paper.

### Partial feedback linearization of the dynamic model

A common method for control of mechanical systems is full-state feedback linearization. This approach is not applicable for snake robots due to the underactuation. However, it is still possible to linearize the dynamics of the actuated DOF of the robot, which is called collocated partial feedback linearization, and can simplify the analysis as well as the control design. A similar approach is considered in [16], but for the sake of completeness, we present the approach here. To this end, we separate the dynamic equations of the robot given by (11) into two subsets by taking $x=\left [q_{a},q_{u}\right ]^{T}\in \mathbb {R}^{N+2}$, with $q_{a}\in \mathbb {R}^{N-1}$ and $q_{u}\in \mathbb {R}^{3}$ which were defined in the subsection describing the geometry of the problem:

(20) $$\begin{array}{@{}rcl@{}}  m_{11} \left(q_{a}\right) \ddot{q}_{a}+m_{12}\left(q_{a}\right)\ddot{q}_{u}+h_{1} (x,\dot{x}) =\psi\in\mathbb{R}^{N-1}  \end{array} $$

(21) $$\begin{array}{@{}rcl@{}} m_{21} \left(q_{a}\right) \ddot{q}_{a}+m_{22}\left(q_{a}\right) \ddot{q}_{u}+h_{2} (x,\dot{x}) =0_{3\times 1}\in \mathbb{R}^{3} \end{array} $$

where $m_{11}\in \mathbb {R}^{(N-1) \times (N-1)}$, $m_{12} \in \mathbb {R}^{(N-1) \times 3}$, $m_{21} \in \mathbb {R}^{3 \times (N-1)}$, and $m_{22}\in \mathbb {R}^{3 \times 3}$ denote the corresponding submatrices of the inertia matrix, and $0_{3\times 1}=\left [0,0,0\right ]^{T}\in \mathbb {R}^{3}$. Furthermore, $h_{1}(x,\dot {x})\in \mathbb {R}^{N-1}$ and $h_{2}(x,\dot {x})\in \mathbb {R}^{3}$ include all the contributions of the Coriolis, centripetal, and friction forces. Moreover, $\psi \in \mathbb {R}^{N-1}$ denotes the non-zero part of the vector of control forces, i.e. $B(x)\tau =\left [\psi,0_{3 \times 1}\right ]^{T}\in \mathbb {R}^{N+2}$. From (21), we have

(22) $$\begin{array}{@{}rcl@{}} \ddot {q}_{u}=-m_{22}^{-1} \left(h_{2} + m_{21}\ddot{q}_{a} \right)\in \mathbb{R}^{3} \end{array} $$

Substituting (22) into (20) yields

(23) $$ \left(m_{11}-m_{12} m_{22}^{-1} m_{21}\right)\ddot{q}_{a}-\left(m_{12} m_{22}^{-1} \right) h_{2}+h_{1}=\psi  $$

For linearizing the dynamics of the directly actuated DOF, we apply the global transformation of the vector of control inputs as

(24) $$ \psi=\left(m_{11}-m_{12} m_{22}^{-1} m_{21}\right)\vartheta-\left(m_{12} m_{22}^{-1}\right) h_{2}+h_{1}  $$

where $\vartheta =\left [\vartheta _{1},\vartheta _{2},\ldots,\vartheta _{N-1}\right ]^{T}\in \mathbb {R}^{N-1}$ is the vector of new control inputs. Consequently, the dynamic model (20)-(21) can be written in the following partially feedback linearized form

(25) $$\begin{array}{@{}rcl@{}} \ddot{q}_{a}&=&\vartheta \in \mathbb{R}^{N-1}  \end{array} $$

(26) $$\begin{array}{@{}rcl@{}} \ddot{q}_{u}&=&\mathcal{D}(x,\dot{x})+\mathcal{C}\left(q_{a}\right)\vartheta\in\mathbb{R}^{3} \end{array} $$

with

(27) $$\begin{array}{@{}rcl@{}} \mathcal{D}(x,\dot{x}) & =& -m_{22}^{-1} \left(q_{a}\right) h_{2} (x,\dot{x})= \left[\,f_{\theta_{N}},f_{x},f_{y}\right]^{T}\in \mathbb{R}^{3}  \end{array} $$

(28) $$\begin{array}{@{}rcl@{}} \mathcal{C}\left(q_{a}\right) & =& -m_{22}^{-1} \left(q_{a}\right)m_{21} \left(q_{a}\right)\\ &=& \left[\beta_{i} \left(q_{a}\right),0,0\right]^{T}\in \mathbb{R}^{3\times {(N-1)}} \end{array} $$

where $\beta _{i}(q_{a}):\mathcal {Q}\rightarrow \mathbb {R}$ is a smooth scalar-valued function. It can be numerically shown that the value of *β*_*i*_ is negative at any configuration $q_{a}\in \mathcal {Q}$. Furthermore, $\,f_{\theta _{N}}$, *f*_*x*_, and *f*_*y*_ denote the friction forces acting on *θ*_*N*_, *p*_*x*_, and *p*_*y*_, respectively ($\,f_{\theta _{N}} $ also contains Coriolis forces besides the friction forces). For the aim of analysis and model-based control design, we write (25)-(26) in a more detailed form:

(29) $$\begin{array}{@{}rcl@{}} \ddot{q}_{a} & = & \vartheta \in \mathbb{R}^{N-1}  \end{array} $$

(30) $$\begin{array}{@{}rcl@{}} \ddot{\theta}_{N} & = & f_{\theta_{N}} (x,\dot{x})+\beta_{i}(q_{a})\vartheta^{i} \in \mathbb{R}  \end{array} $$

(31) $$\begin{array}{@{}rcl@{}} \ddot{p}_{x} & = & f_{x} (x,\dot{x}) \in \mathbb{R}  \end{array} $$

(32) $$\begin{array}{@{}rcl@{}} \ddot{p}_{y} & = & f_{y} (x,\dot{x})\in \mathbb{R} \end{array} $$

where the summation convention is applied in (30), and henceforth, to all the equations which contain repeated upper-lower indices (i.e. whenever an expression contains a repeated index, one as a subscript and the other as a superscript, summation is implied over this index [26]). The dynamical system (29)-(32) is in the form of a control-affine system with drift. In particular, the term

(33) $$ \mathcal{A}(x,\dot{x})=\left[\dot q_{a}, \dot{q}_{u},0_{(N-1) \times 1},\mathcal{D}(x,\dot{x})\right]^{T}\in \mathbb{R}^{2N+4}  $$

is called the drift vector field, which specifies the dynamics of the robot when the control input is zero. Furthermore, the columns of the matrix

(34) $$\begin{array}{@{}rcl@{}}  \mathcal{B}\left(q_{a}\right)\,=\, \left[\!\!\! \begin{array}{cc} 0_{(N+2) \times(N-1)} \\ I_{N-1} \\ \left[\beta_{1} \left(q_{a}\right),\ldots,\beta_{N-1} \left(q_{a}\right)\right] \\ 0_{2 \times (N-1)} \end{array} \!\!\!\right]\!\! \in \mathbb{R}^{(2N+4) \times (N-1)} \end{array} $$

are called the control vector fields, which enable us to control the internal configuration and consequently the orientation and the position of the robot in the plane.

#### **Remark2**.

The last two rows of the control vectors in (34) are composed of zero elements. This implies that the control forces have no direct effect on the dynamics of the position of the CM of the robot, i.e. (31)-(32). Furthermore, the dynamics of the position of the CM are coupled with the dynamics of the directly actuated shape variables *q*_*a*_, i.e. (29), only through the friction forces. Accordingly, in the absence of the friction forces, the linear momentum of the robot is a conserved quantity, and the position of the CM of the robot is not controllable.

## Control design objectives and the track-follow problem formulation

In this section, we state our control design objectives which will be followed throughout the remaining sections of the paper. In particular, we stress that for a complex mobile multi-link robotic structure such as a snake robot, formulating a pure path following, trajectory tracking, or maneuvering problem is unusual (for definitions of these problem formulations, see [27]). This is due to the fact that for a part of the state variables of the system (particularly the shape variables and the head angle), it is most natural to formulate the control problem as a trajectory tracking problem, while for the other state variables (particularly the position of the CM), we may formulate the problem as a path following or a maneuvering one.

To formulate a combinational *track-follow* problem for the snake robot, which we define as a trajectory tracking formulation for a subset of the state variables, together with a path following formulation for the remaining subset, we introduce the error variable for the *i*th joint of the robot as

(35) $$ y_{i}=q_{i}-\Phi_{i}  $$

where *i*∈{1,…,*N*−1}, and $\Phi _{i}\in \mathbb {R}$ denotes a function that defines the reference trajectory for the *i*th joint which will be chosen through the control design in the next section. The head angle error is defined as

(36) $$ y_{N}=\theta_{N}-\Phi_{N}  $$

where $\Phi _{N} \in \mathbb {R}$ denotes the reference head angle for the robot.

We divide the control objectives into three main parts. In the first part, the goal is to make the shape variables of the robot track given bounded smooth time-varying references, i.e. asymptotic trajectory tracking problem, such that

(37) $$ \lim\limits_{t \to \infty}\| y_{i} (t)\|=0  $$

for all *i*∈{1,…,*N*−1}. Furthermore, we seek to control the head angle of the robot. The second part of the control objective is thus to make the head angle of the robot track a desired head angle such that

(38) $$ \lim\limits_{t \to \infty}\| y_{N} (t)\|=0  $$

Moreover, we define a desired straight path that we want the CM of the snake robot to follow. This is defined as a smooth one-dimensional manifold $\mathcal {P}\subset \mathbb {R}^{2}$, with coordinates in the *x*-*y* plane given by the pair (*p*_*xd*_,*p*_*yd*_), which are parameterized by a scalar time-dependent variable *Θ*(*t*) as

(39) $$ \mathcal{P}=\left\{\left(p_{xd}(\Theta),p_{yd} (\Theta)\right) \in \mathbb{R}^{2}: \Theta\geq 0\right\}  $$

We define the vector of the path following error variables for the position of the CM of the robot as $\tilde {p}=\left [p_{x}(t)-p_{\textit {xd}}(\Theta),p_{y}(t)-p_{\textit {yd}}(\Theta)\right ]^{T}\in \mathbb {R}^{2}$. Subsequently, the third part of the control objectives is defined as practical convergence (see, e.g. [4]) of the position of the CM of the robot to the desired path such that

(40) $$ \lim\limits_{t \to \infty} \sup\| \tilde{p} (t)\|\leq \varepsilon  $$

where $\varepsilon \in \mathbb {R}_{>0}$ is an arbitrary positive scalar. Moreover, we require that $\dot \Theta (t)\geq 0$ and ${\lim }_{t \to \infty } \Theta (t) =\infty $ (forward motion along the path), and boundedness of the states of the controlled system.

## Path following control with virtual holonomic constraints

The idea of virtual holonomic constraints is particularly a useful concept for control of oscillations (see, e.g. [20-24]). We will in this section show how this approach can be used to solve the path following control problem of snake robots. In particular, we will show how, by designing the joint reference trajectories in (35) using virtual holonomic constraints and by combining this with virtual holonomic constraints motivated by line-of-sight (LOS) guidance for the head angle in (36), we are able to solve the path following control problem, i.e. achieving (40). Our main motivation for using this approach is the fact that while performing the gait pattern lateral undulation which consists of fixed periodic body motions, all the solutions of the snake robot dynamics have inherent oscillatory behaviour. Moreover, we will show how this behaviour can be analytically and *constructively* controlled based on virtual holonomic constraints. In particular, we use the word ‘constructive’ in the sense that through the feedback action, we shape the dynamics of the system such that it possesses the desired structural properties, i.e. positive invariance and exponential stability of an appropriately defined constraint manifold. To this end, we define a constraint manifold for the system, and we design the control input of (29) to exponentially stabilize the constraint manifold. The geometry of this manifold is defined based on specified geometric relations among the generalized coordinates of the system which are called virtual holonomic constraints. In particular, we call them virtual constraints because they do not arise from a physical connection between two variables but rather from the actions of a feedback controller [20].

### Trajectory planning by virtual holonomic constraints

Virtual holonomic constraints are specified through *C*^1^ coordinate-dependent functions $\Phi _{i}:\mathcal {Q}\to \mathbb {R}$ which are called the constraint functions, in the relations of the form *Φ*_*i*_(*x*)=0, which can be enforced through the feedback action. In particular, for the snake robot, we define a vector-valued function

(41) $$ \Phi =\left[\Phi_{1},\ldots,\Phi_{N} \right]^{T}\in \mathbb{R}^{N}  $$

in which every element defines one constraint function for the corresponding angular coordinate of the system.

At this point, we augment the state vector of the system with three new states that in the following will be used in the control design. The introduction of these new variables to the state vector of the system, which will be used as constraint variables, is inspired by the notion of dynamic virtual holonomic constraints [21], i.e. virtual holonomic constraints which depend on the solutions of a dynamic compensator. The idea is to make the virtual holonomic constraints to depend on the variations of a dynamic parameter, which is used for controlling the system on the constraint manifold. The purpose of these additional states is explained below.

1. We introduce two new states $\left [\phi _{o},\dot \phi _{o}\right ]^{T}\in \mathbb {R}^{2}$ where the second-order time derivative of *ϕ*_*o*_ will be used as an additional control input that drives the snake robot towards the desired path by modifying the orientation of the robot in accordance with a path following guidance law.
2. In the previous section, we defined the control objective for the joints and the head angle of the robot as a trajectory tracking problem. However, it is known that holonomic constraints are coordinate-dependent equality constraints of the form *Φ*_*i*_(*x*)=0, where *Φ*_*i*_ is a time-independent function [25]. Thus, we remove this explicit time dependency from the reference joint trajectories by augmenting the state vector of the system with a new variable *η*, with $\dot {\eta }=2\pi /T$ and *η*(0)=0, where *T* denotes the period of the cyclic motion of the shape variables of the robot.
  Subsequently, we denote the augmented coordinate vector of the system by
  (42) $$ \hat x=\left[q_{1},\ldots,q_{N-1},\theta_{N},p_{x},p_{y},\phi_{o},\eta \right]^{T}\in \mathbb{R}^{N+4}  $$
  and the corresponding augmented state space by $T\hat {\mathcal {Q}}$.

### Virtual holonomic constraints for the joint angles

A fundamental work in the area of snake robots was presented by Hirose [5]. In this work, Hirose considers empirical studies of biological snakes to derive a mathematical approximation of the most common gait pattern among biological snakes, known as lateral undulation. In particular, the shape of a snake conducting lateral undulation can be described by a planar curve (the serpenoid curve) with coordinates in the *x*-*y* plane along the curve at arc length *s* given by

(43) $$\begin{array}{@{}rcl@{}} x(s) = {\int_{0}^{s}} \cos\left(a\cos(bz)+cz\right)dz  \end{array} $$

(44) $$\begin{array}{@{}rcl@{}} y(s) = {\int_{0}^{s}} \sin(a\cos(bz)+cz)dz \end{array} $$

where *a*, *b*, and *c* are positive scalars. Locomotion of a snake-like structure in accordance with the serpenoid curve, i.e. lateral undulation, is achieved if the joints of the robot move according to the reference joint trajectories in the form of a sinusoidal function with specified amplitude, frequency, and phase shift. In particular, using the foregoing defined new states, we define a constraint function for the *i*th joint of the snake robot by

(45) $$ \Phi_{i} = \alpha \sin\left(\eta + (i-1)\delta\right) + \phi_{o}  $$

where *i*∈{1,…,*N*−1}, *α* denotes the amplitude of the sinusoidal joint motion, and *δ* is a phase shift that is used to keep the joints *out of phase*. Moreover, *ϕ*_*o*_ is an offset value that is identical for all of the joints. It was illustrated in [16] how the offset value *ϕ*_*o*_ affects the orientation of the snake robot in the plane. Building further on this insight, we consider the second-order time derivative of *ϕ*_*o*_ in the form of a dynamic compensator, which will be used to control the orientation of the robot. In particular, through this control term, we modify the orientation of the robot in accordance with a reference orientation. This will be done by adding an offset angle to the reference trajectory of each joint. We will show that this will steer the position of the CM of the robot towards the desired path. The constraint function (45) is dynamic, since it depends on the solution of a dynamic compensator.

### Virtual holonomic constraint for the head link angle

In this subsection, we define a constraint function for the head angle of the robot. In particular, we use a line-of-sight (LOS) guidance law as the reference angle for the head link. LOS guidance is a much-used method in marine control systems (see, e.g. [27]). In general, guidance-based control strategies are based on defining a reference heading angle for the vehicle through a guidance law and designing a controller to track this angle [27]. Motivated by marine control literature, in [17] based on a simplified model of the snake robot, using cascade systems theory, it was proved that if the heading angle of the snake robot was controlled to the LOS angle, then also the position of the CM of the robot would converge to the desired path. We will show that a similar guidance-based control strategy can successfully steer the robot towards the desired path. However, we perform the model-based control design based on a more accurate model of the snake robot which does not contain the simplifying assumptions of [17] which are valid for small joint angles.

To define the guidance law, without loss of generality, we assign the global coordinate system such that the global *x*-axis is aligned with the desired path. Consequently, the position of the CM of the robot along the *y*-axis, denoted by *p*_*y*_, defines the shortest distance between the robot and the desired path, often referred to as the *cross-track error*. In order to solve the path following problem, we use the LOS guidance law as a virtual holonomic constraint, which defines the desired head angle as a function of the cross-track error as

(46) $$ \Phi_{N}=-\tan^{-1}\left(\frac{p_{y}}{\Delta}\right)  $$

where *Δ*>0 is a design parameter known as the look-ahead distance. The idea is that steering the head angle of the snake robot such that it is headed towards a point located at a distance *Δ* ahead of the robot along the desired path will make the snake robot move towards the path and follow it.

### Defining a constraint manifold

We collect all the foregoing defined constraint functions in the following vector-valued function

(47) $$ \begin{aligned} \Phi =&\, \left[\vphantom{\frac{p_{y}}{\Delta}}\alpha \sin(\eta)+\phi_{o}, \ldots, \alpha\sin\left(\eta + (N-1)\delta\right)\right.\\ &\left.\;+\phi_{o}, \tan^{-1}\left(\frac{p_{y}}{\Delta}\right)\right]^{T} \in \mathbb{R}^{N} \end{aligned}  $$

For trajectory planning using virtual holonomic constraints, we define the constraint manifold associated with the constraint functions (47) as

(48) $$ \begin{aligned} \Gamma =&\, \left\{{\vphantom{\frac{1}{2}}}\left(\hat{x},\dot{\hat{x}}\right)\in T\hat{\mathcal{Q}}: q_{i}=\Phi_{i}\left(\eta,\phi_{o}\right),\theta_{N}=\Phi_{N}\left(p_{y}\right), \right. \\ &\, \left.\dot{q}_{i}=\dot{\eta}\frac{\partial \Phi_{i}}{\partial \eta}+ \dot{\phi}_{o}\frac{\partial \Phi_{i}}{\partial \phi_{o}}, \dot{\theta}_{N}=\dot{p}_{y}\frac{\partial \Phi_{N}} {\partial p_{y}} \right\} \end{aligned}  $$

where *i*∈{1,…,*N*−1}. The constraint manifold (48) is a six-dimensional submanifold of $ \hat {\mathcal {Q}}$, since we have three different constraint variables, i.e. *η*,*ϕ*_*o*_,*p*_*y*_. The goal of the control input is to enforce the virtual holonomic constraints (47), by making *Γ* exponentially stable for the closed-loop system and thereby achieving the control objectives (37)-(38). To this end, we define the elements of a controlled output vector $y\in \mathbb {R}^{N}$ for the system (29)-(32) as the difference between the angular coordinates and their corresponding constraint functions as

(49) $$ \begin{aligned} y=&\,\left[q_{1}-\Phi_{1} \left(\eta,\phi_{o}\right),\ldots,q_{N-1}\right.\\ &\left.\,-\,\Phi_{N-1} \left(\eta,\phi_{o}\right),\theta_{N}-\Phi_{N} \left(p_{y}\right)\right]^{T}\in \mathbb{R}^{N} \end{aligned}  $$

We will achieve our control design objectives which we defined in the previous section, by designing the control inputs *𝜗* and $\ddot {\phi }_{o}$ such that $\left (y_{i},\dot {y}_{i}\right)\to (0,0)$ for all *i*∈{1,…,*N*}. To this end, we first need to ensure that the given relations in (47) are stabilizable, i.e. a suitable choice of feedback can make the constraint manifold exponentially stable for the closed-loop system. For simplicity of notation, we denote the following differentials:

(50) $$\begin{array}{@{}rcl@{}}  d \Phi_{i} &=&\dot{\eta}\frac{\partial \Phi_{i}}{\partial \eta}+\dot{\phi}_{o}\frac{\partial \Phi_{i}} {\partial \phi_{o}} \end{array} $$

(51) $$\begin{array}{@{}rcl@{}} d \Phi_{N} & =&\dot{p}_{y} \frac{\partial \Phi_{N}}{\partial p_{y}} \end{array} $$

(52) $$\begin{array}{@{}rcl@{}} d^{2} \Phi_{i} &=&\ddot{\eta}\frac{\partial \Phi_{i}}{\partial \eta}+\dot \eta^{2} \frac{\partial^{2} \Phi_{i}} {\partial \eta^{2}}+\ddot{\phi}_{o}\frac{\partial \Phi_{i}}{\partial \phi_{o}}+ \dot{\phi}_{o}^{2}\frac{\partial^{2} \Phi_{i}}{\partial {\phi_{o}^{2}}} \end{array} $$

(53) $$\begin{array}{@{}rcl@{}} d^{2} \Phi_{N} & =&\ddot{p}_{y} \frac{\partial \Phi_{N}}{\partial p_{y}}+\dot {p_{y}^{2}} \frac{\partial^{2} \Phi_{N}}{\partial {p_{y}^{2}}} \end{array} $$

where *i*∈{1,…,*N*−1}. The Lie derivative of (49) along the solutions of (29)-(32) is of the form

(54) $$  \dot{y}=\left[\dot{q}_{1}-d \Phi_{1},\ldots,\dot{q}_{N-1}-d \Phi_{N-1},\dot{\theta}_{N} -d \Phi_{N}\right]^{T} \in \mathbb{R}^{N}  $$

which lacks the control inputs. The Lie derivative of (54) along the solutions of (29)-(32) is of the form

(55) $$\begin{array}{*{20}l} \ddot{y}=&\,\left[\vartheta_{1}-d^{2} \Phi_{1},\ldots,\vartheta_{N-1}\right.\\ &\left.-\, d^{2} \Phi_{N-1},\, f_{\theta_{N}} +\beta_{i} \vartheta^{i}-d^{2} \Phi_{N}\right]^{T}\in \mathbb{R}^{N } \end{array} $$

which contains the control inputs. Consequently, the controlled output vector (49) yields a well-defined vector relative degree {2,2,…,2} everywhere on the constraint manifold *Γ*. The virtual holonomic constraints satisfying this vector relative degree condition are called regular, and regular constraints are always feasible [22], i.e. there exists a smooth feedback such that *Γ* is positively invariant for the closed-loop system. Furthermore, regular constraints in parametric form (47) are always stabilizable [21].

The well-defined vector relative degree {2,2,…,2} on *Γ* implies that the system (29)-(32) with the controlled output function (47) is input-output feedback linearizable. Consequently, we can stabilize *Γ* with an input-output feedback linearizing controller.

### Output regulation via input-output linearization

In this subsection, we will derive a control law for (29) such that the constraint manifold (48) with the constraint functions defined in (47) is globally exponentially stable for the closed-loop system. In particular, we use input-output linearization to stabilize the constraint manifold (48).

To stabilize *Φ*_*i*_(*η*,*ϕ*_*o*_) for the *i*th joint, i.e. to make $(y_{i},\dot {y}_{i})\to (0,0)$ for all *i*∈{1,…,*N*−1}, we define an exponentially stabilizing joint control law. The second-order time derivative of the *i*th joint tracking error, i.e. the *i*th element of (55), is of the form

(56) $$ \ddot{y}_{i}=\vartheta_{i}-d^{2} \Phi_{i}  $$

We define the control input for the *i*th joint in (29) as

(57) $$ \vartheta_{i}=d^{2} \Phi_{i}-k_{p} y_{i}-k_{d} \dot{y}_{i}  $$

where *k*_*p*_>0 and *k*_*d*_>0 are the joint controller gains. These gains are chosen similar for all the joints since the links have similar inertial parameters. Inserting (57) into (56) yields

(58) $$ \ddot{y}_{i}+k_{d} \dot{y}_{i}+k_{p} y_{i}=0  $$

The tracking error dynamics of the *i*th joint angle (58) clearly has a globally exponentially stable equilibrium at the origin $\left (y_{i},\dot {y}_{i})=(0,0\right)$, which implies that every *i*th control input (57) exponentially stabilizes the constraint manifold for the *i*th joint, and the control objective (37) is achieved.

In the following, we aim to stabilize the constraint manifold for the head angle, i.e. to make $\left (y_{N},\dot {y}_{N}\right)\to (0,0)$. The head angle error corresponds to the *N*th element of the controlled output vector (49), and its second-order time derivative (i.e. the head angle error dynamics) is given by

(59) $$ \ddot{y}_{N}=f_{\theta_{N}} +\beta_{i} \vartheta^{i}-d^{2} \Phi_{N}  $$

Inserting *𝜗*_*i*_ from (57) into (59) gives

(60) $$  \ddot{y}_{N}=f_{\theta_{N}}+\sum\limits_{i=1}^{N-1}\beta_{i} \left(d^{2} \Phi_{i}-k_{p} y_{i}-k_{d} \dot{y}_{i} \right)-d^{2} \Phi_{N}  $$

which is equivalent to

(61) $$ {\fontsize{9}{10}\begin{aligned} \ddot{y}_{N}=f_{\theta_{N}} + \sum\limits_{i=1}^{N-1}\beta_{i} &\,\left(\ddot{\eta} \frac{\partial \Phi_{i}} {\partial \eta}+\dot{\eta}^{2} \frac{\partial^{2} \Phi_{i}}{\partial \eta^{2}}+\ddot{\phi}_{o} \frac{\partial\Phi_{i}}{\partial \phi_{o}}\right.\\ &\;\;\left.+\dot{\phi}_{o}^{2} \frac{\partial^{2} \phi_{i}}{\partial {\phi_{o}^{2}}}-k_{p} y_{i}-k_{d} \dot{y}_{i}\right)-d^{2} \Phi_{N} \end{aligned}}  $$

For simplicity of notation, we denote the constraint function for the *i*th joint angle of the robot by *Φ*_*i*_=*S*_*i*_+*ϕ*_*o*_, where *S*_*i*_=*α* sin(*η*+(*i*−1)*δ*). Subsequently, based on the specified constraint functions in (47), i.e. since $\ddot {\eta }=0$ and $\frac {\partial ^{2} \phi _{i}}{\partial {\phi _{o}^{2}}}=0$, we may write (61) as

(62) $$ \ddot{y}_{N} = f_{\theta_{N}} + \sum\limits_{i=1}^{N-1}\beta_{i} \left(-\dot{\eta}^{2} S_{i}+\ddot{\phi}_{o} -k_{p} y_{i}-k_{d} \dot{y}_{i}\right)-d^{2} \Phi_{N}  $$

In order to stabilize the constraint function *Φ*_*N*_(*p*_*y*_) for the head angle, we define the second-order time derivative of the augmented coordinate *ϕ*_*o*_ in the form of a dynamic compensator as

(63) $$ {\begin{aligned} \ddot{\phi}_{o} =& \\ &\left(\sum\limits_{i=1}^{N-1}\beta_{i}\right)^{-1} \left(\sum\limits_{i=1}^{N-1}\beta_{i} \left(\dot{\eta}^{2} S_{i}+ k_{p} y_{i}+k_{d} \dot{y}_{i}\right)\right.\\ &\left.\;+\;d^{2} \Phi_{N} -f_{\theta_{N}}- k_{p,\theta_{N}}y_{N}-k_{d,\theta_{N}} \dot{y}_{N}\vphantom{\sum\limits_{i=1}^{N-1}\beta_{i}} \right) \end{aligned}}  $$

where $k_{p,\theta _{N}}>0$ and $k_{d,\theta _{N}}>0$ are the head angle controller gains. Notice that since *β*_*i*_ is negative-valued in any configuration, (63) is globally well-defined. Through numerical simulations, we show that the states of the dynamic compensator (63), i.e. $(\phi _{o}, \dot {\phi }_{o})$, remain bounded. By inserting (63) into (62), the error dynamics of the head angle takes the form

(64) $$ \ddot{y}_{N}+k_{d,\theta_{N}} \dot{y}_{N}+k_{p,\theta_{N}} y_{N}=0  $$

which clearly has a globally exponentially stable equilibrium at the origin $(y_{N},\dot {y}_{N})=(0,0)$. Consequently, we have that $(y_{N},\dot {y}_{N})\to (0,0)$ from any initial condition, and the control objective (38) will be achieved.

Finally, we conjecture that while the output trajectories are evolving on the constraint manifold (48), the internal dynamics (31)-(32), which has the form

(65) $$ \ddot{p}_{x}=f_{x} \left(\Phi,p_{x},p_{y},\Phi^{\prime},\dot{p}_{x},\dot{p}_{y}\right)  $$

(66) $$ \ddot{p}_{y}=f_{y} \left(\Phi,p_{x},p_{y},\Phi^{\prime},\dot{p}_{x},\dot{p}_{y}\right)  $$

converge to and follow the desired planar path. Analytically investigating the convergence of the snake robot position to the desired path is a topic of future work. As a preliminary support of this conjecture, we provide simulation and experimental results which show that the snake robot successfully converges to and follows the desired path.

## Simulation results

**Figure 2:**
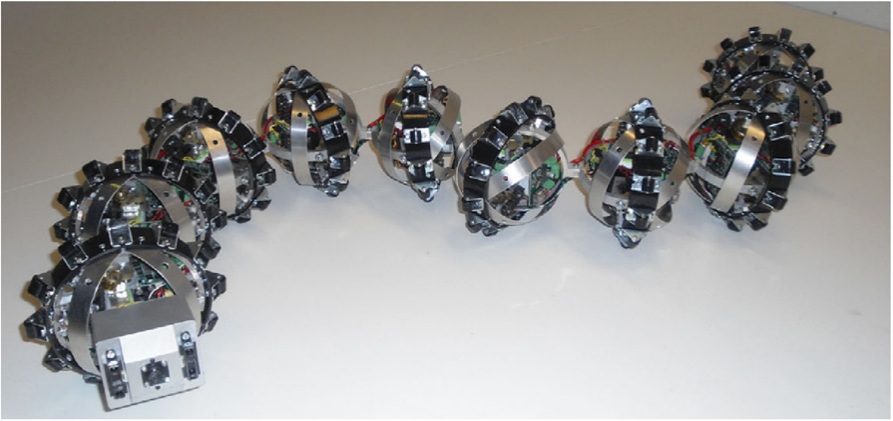
**An illustration of the experimental setup.** Snake robot Wheeko was used for the experiments.

**Figure 3:**
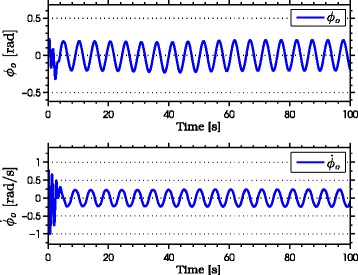
**The states of the dynamic compensator in simulations.** The states of the dynamic compensator remain bounded.

**Figure 4:**
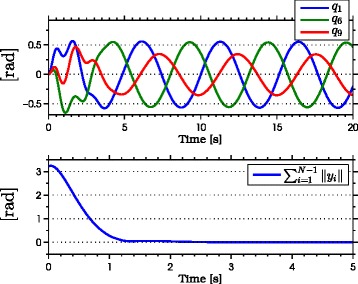
**Joint angles tracking the reference joint angles in simulations.** The joints of the robot track the sinusoidal motions (above). The joint tracking errors converge exponentially to zero (below).

**Figure 5:**
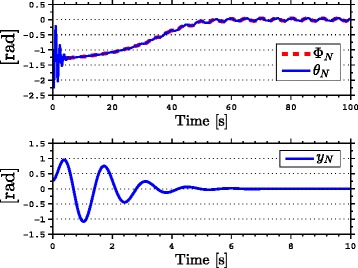
**Head angle tracking error in simulations.** The head angle tracking error converges exponentially to zero.

**Figure 6:**
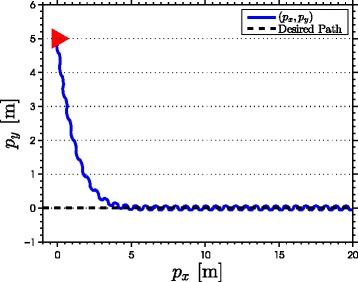
**The motion of the center of mass in the** ***x*** **-** ***y*** **plane in simulations.** The position of the CM of the robot (blue) converges to and follows the desired straight line path (the *x*-axis).

**Figure 7:**
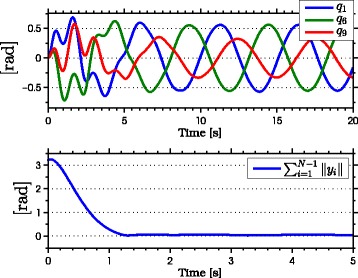
**Joint angles tracking the reference joint angles in simulations in the presence of measurement noise.** The joints of the robot track the sinusoidal motions in the presence of measurement noise (above). The joint tracking errors converge exponentially to a neighbourhood of zero in the presence of measurement noise (below).

In this section, we present simulation results which illustrate the performance of the proposed path following controller. We considered a snake robot with *N*=11 links, *m*=1 kg, *l*=0.07 m, and *J*=0.0016 kg m ^2^ (Figure 2). The friction coefficients were *c*_*n*_=10 and *c*_*t*_=1. The parameters of the joint constraint functions (45) were *α*=*π*/6 rad, *η*=70*πt*/180 rad, and *δ*=36*π*/180 rad. The controller gains in (57) and (63) were tuned as *k*_*p*_=10, *k*_*d*_=5, $k_{p,\theta _{N}}=20$, $k_{d,\theta _{N}}=1$, and *Δ*=1.4 m. In order to calculate $\dot {\Phi }_{N}$ and $\ddot {\Phi }_{N}$, we employed the approach taken in [27] by passing *Φ*_*N*_ through a low-pass filter of the form

(67) $$ \dot{\Omega}= \left[ \begin{array}{cc} 0 & 1 \\ -{\omega_{n}^{2}} & -2\psi_{f}\omega_{n} \\ \end{array} \right] \Omega+ \left[ \begin{array}{cc} 0 \\ {\omega_{n}^{2}} \\ \end{array} \right] \Phi_{N}  $$

with natural frequency *ω*_*n*_=*π*/2 rad, damping ratio *ψ*_*f*_=1, and initial condition *Ω*(0)=[0,0]^*T*^. As seen from the simulation results which are presented in Figures 3, 4, 5, 6, 7, the snake robot successfully converges to and follows the desired path. In particular, Figure 3 shows that the solutions of the dynamic compensator (63) remain bounded. Figure 4 shows that the joint angles track the reference angles provided by the constraint functions (45), while the tracking errors converge exponentially to zero. Figure 5 shows that the head angle tracks the reference head angle provided by the constraint function (46), while the tracking error converges to zero exponentially fast. Finally, Figure 6 shows that the CM of the robot converges to and follows the desired straight path. Moreover, in order to show the performance of the proposed tracking control law (57) in the presence of angular position measurement noise, we subjected every *i*th joint angle *q*_*i*_ to an additive noise by using Matlab function *randn*() which generates normally distributed pseudorandom numbers that can be considered as measurement noise for the joint angles. In particular, we added *randn*(1) to each joint angle *q*_*i*_ in each integration step. The result of the simulation is presented in Figure 7, which shows that the joint tracking errors converge to a very small neighbourhood of zero in the presence of measurement noise.

## Experimental results

In this section, we present results from an experimental investigation of the real-time performance of the proposed control strategy using a mechanical snake robot.

### Experimental setup

The experiment was carried out using the snake robot Wheeko [16]. The robot, which is shown in Figure 2, has 10 identical joint modules, i.e. *N*=11 links. Each joint module is equipped with a set of passive wheels which give the robot anisotropic ground friction properties during motion on flat surfaces. The wheels are able to slip sideways and thus do not introduce nonholonomic velocity constraints in the system. Each joint is driven by a Hitec servo motor (HS-5955TG; Hitec RCD USA, Inc., Poway, CA, USA), and the joint angles are measured using magnetic rotary encoders. The motion of the snake robot was measured using a camera-based motion capture system from OptiTrack of type Flex 13 (NaturalPoint, Inc., Corvallis, OR, USA). The system consists of 16 cameras which are sampled at 120 frames per second and which allow reflective markers to be tracked on a submillimetre level. During the experiment, reflective markers were mounted on the head link of the snake robot in order to measure the position (*p*_*x*,*N*_,*p*_*y*,*N*_) and orientation (*θ*_*N*_) of the head. These measurements were combined with the measured joint angles (*q*_1_,…,*q*_*N*−1_) of the snake robot in order to measure the absolute link angles (1) and the position of the CM (*p*_*x*_,*p*_*y*_) of the robot. In order to obtain the derivatives of the reference head angle (46), we used the same technique as in the simulations, i.e. passing *Φ*_*N*_ through a low-pass filter of the form (67). The parameters of the low-pass filter were set to *ω*_*n*_=*π*/2 and *ψ*_*f*_=1.

In the following, we elaborate on a few adjustments that were made in the implemented path following controller in order to comply with the particular properties and capabilities of the physical snake robot employed in the experiment. We conjecture that these adjustments only marginally affected the overall motion of the robot. The successful path following behaviour of the robot demonstrated below supports this claim. Since the experimental setup only provided measurements of the joint angles and the position and orientation of the head link, we chose to implement the joint controller in (57) as

(68) $$ \vartheta_{i} = -k_{p}y_{i}  $$

where *i*∈{1,…,10}. We conjecture that eliminating the joint angular velocity terms from (57) did not significantly change the dynamic behaviour of the system since the joint motion was relatively slow during the experiment. The main consequence of excluding the velocity terms from (57) is that we potentially introduce a steady-state error in the tracking of the joint angles. Consequently, since with the joint control law (68) the derivative terms in (63) are identically zero, they need not to be linearized in the head angle dynamics by the dynamic compensator. As the result, we implemented the dynamic compensator of the form

(69) $$\begin{array}{*{20}l}  \ddot{\phi}_{o} =&\, \left(\sum\limits_{i=1}^{N-1}\beta_{i}\right)^{-1} \left(-f_{\theta_{N}}+d^{2} \Phi_{N}-k_{p,\theta_{N}}y_{N}-k_{d,\theta_{N}} \dot{y}_{N} \right)\\ & -\, k_{p}\phi_{o} -k_{d}\dot{\phi}_{o} \end{array} $$

where the controller gains were $k_{p,\theta _{N}}=20$, $k_{d,\theta _{N}}=1$, *k*_*p*_=10, and *k*_*d*_=5. We saturated the joint angle offset *ϕ*_*o*_ according to *ϕ*_*o*_∈[−*π*/6,*π*/6], in order to keep the joint reference angles within reasonable bounds w.r.t the maximum allowable joint angles of the physical snake robot. Moreover, from Figure 2, it can be seen that the head link of the physical snake robot does not touch the ground since the ground contact points occur at the location of the joints. As a results, we implemented (69) with $\,f_{\theta _{N}} \equiv 0$. The solutions of the dynamic compensator (69) were obtained by numerical integration in *LabVIEW* which was used as the development environment.

We chose the look-ahead distance of the path following controller as *Δ*=1.4 m. The initial values for the configuration variables of the snake robot were *q*_*i*_=0 rad, *θ*_*N*_=−*π*/2 rad, *p*_*x*_=0.3 m, and *p*_*y*_=1.7 m, i.e. the snake robot was initially headed towards the desired path (the *x*-axis), and the initial distance from the CM to the desired path was 1.7 m. Furthermore, the parameters of the constraint functions for the joint angles, i.e. (45), were *α*=*π*/6, *η*=70*πt*/180, and *δ*=36*π*/180, while the ground friction coefficients were *c*_*t*_=1 and *c*_*n*_=10 (i.e identical to the simulation parameters).

### Results and discussion

**Figure 8:**
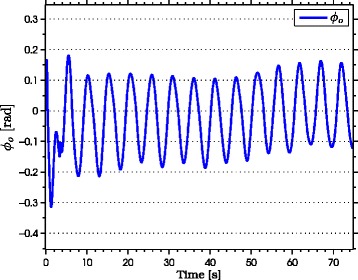
**The solution of the dynamic compensator in experiments.** The solution of the dynamic compensator remains bounded during the experiments.

**Figure 9:**
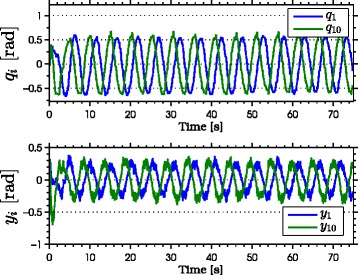
**Joint angles tracking the reference joint angles during the experiments.** The joints of the robot track the sinusoidal motions (above). The tracking errors converge to a neighbourhood of zero during the experiment (below).

**Figure 10:**
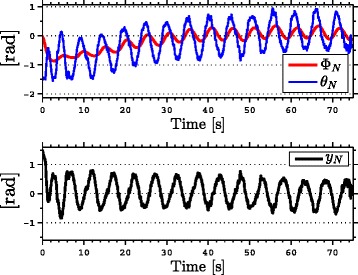
**Head angle tracking error during the experiments.** The head angle tracks the reference head angle (above). The head angle tracking error converges to a neighbourhood of zero (below).

**Figure 11:**
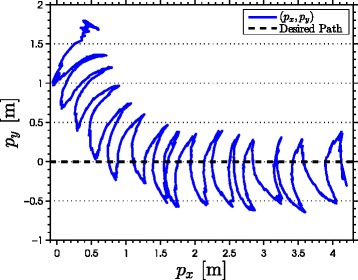
**The motion of the center of mass in the** ***x*** **-** ***y*** **plane during the experiments.** The position of the CM of the robot (blue) converges to and follows the desired straight path (the *x*-axis).

**Figure 12:**
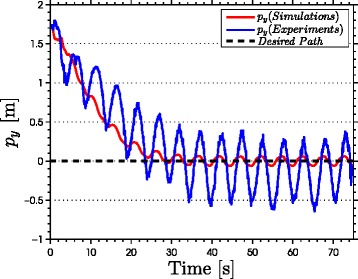
**Comparison between experiments and simulations.** Comparison of the convergence of the cross-track error during simulations (red) and experiments (blue).

**Figure 13:**
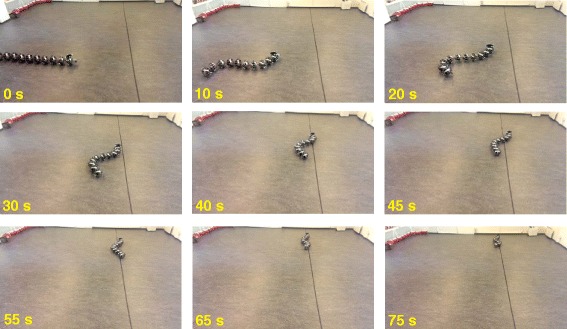
**An image of the motion of the robot during the experiments.** The robot converges to and follows the desired path.

The results of the experiments are illustrated in Figures 8, 9, 10, 11, 12, 13. In particular, Figure 8 shows that the solution of the dynamic compensator remained bounded. Figure 9 shows that the joints of the robot tracked the sinusoidal reference angles provided by the constraint functions (45) and that the tracking error converged to a neighbourhood of the origin. As discussed above, this is probably due to the modification of the joint controller (68) due to the lack of velocity measurements in the lab. Figure 10 shows that the head angle of the robot tracked the reference head angle defined by the constraint function (46) and that the tracking error converged to a neighbourhood of the origin. Figure 11 shows the motion of the CM of the robot in the *x*-*y* plane, which converged to and followed the desired path. Figure 12 compares the motion of the CM during the simulation and the experiment, which were performed using the same controller parameters in order to obtain comparable results. In particular, from Figure 12, it can be seen that the physical snake and the simulated snake follow almost the same path. However, due to precise measurement and a more accurate joint control law for the simulated snake, the path following error converges to a smaller neighbourhood of the origin. Figure 13 shows screenshots from a video recording of the experiment.

## Conclusions

This paper has considered path following control of planar snake robots by using virtual holonomic constraints. The equations of motion of the snake robot were derived using a Lagrangian framework. We then introduced virtual holonomic constraints that defined the geometry of a constraint manifold for the robot. We showed that the constraint manifold can be made positively invariant by a suitable choice of feedback, and we designed an input-output feedback linearizing control law to exponentially stabilize the constraint manifold for the system. We presented simulation and experimental results which validated the theoretical design. In particular, the robot successfully converged to and followed a desired straight path.

As a topic of future work, we aim to prove the practical stability of the desired path with the proposed control approach. Furthermore, a formal proof for boundedness of the solutions of the dynamic compensator remains as a topic of future work. Moreover, application of the proposed control strategy for more complex paths such as curved paths, using different path following guidance laws, remains as a topic of future work.
